# Orexin-A and Periosteal Pressure Sensitivity during acute mental and physical stress: a randomized cross-over study

**DOI:** 10.3389/fnins.2026.1923282

**Published:** 2026-10-07

**Authors:** Nanna Ørsted, Karolina Sikorska, Søren Ballegaard, Katrine Qvortrup

**Affiliations:** 1 Department of Chemistry, Technical University of Denmark, Kongens Lyngby, Denmark; 2 Department of Health Technology, Technical University of Denmark, Kongens Lyngby, Denmark; 3 Ballegaard Ltd., Skodsborg, Denmark

**Keywords:** autonomic nervous system, cardiovascular regulation, orexin-A, Periosteal Pressure Sensitivity, stress

## Abstract

**Background:**

Objective physiological markers reflecting central autonomic regulation remain limited. The orexin (hypocretin) system, localized in the lateral hypothalamus, integrates arousal, metabolic state, cardiovascular control, homeostatic control, and stress responsiveness. Periosteal Pressure Sensitivity (PPS) at the sternum has been proposed as a peripheral marker for central sympathetic activation. The present study examined for the first time the association between PPS and salivary orexin-A during experimentally induced mental and physical stress and subsequent recovery.

**Methods:**

In this randomized cross-over study, 20 healthy female participants (20–28 years) completed two experimental procedures in randomized order: Maastricht Acute Stress Test (MAST) and Moderate-Intensity Interval Training (MIIT). PPS, systolic blood pressure, heart rate, Pressure-Rate Product (i.e., systolic blood pressure × heart rate) and salivary orexin-A were measured at baseline, immediately post-procedure, as well as 15 and 30 mins into recovery. MIIT results were analyzed both for the full sample (*n* = 20) and for the participants who fulfilled the protocol-defined cardiovascular response criterion (*n* = 12).

**Results:**

MAST did not induce significant baseline-to-post-stress changes in PRP, PPS, or salivary orexin-A, although PRP decreased significantly during early recovery from S2 to S3 (*p* = 0.045). A significant negative correlation was observed between ΔPPS and ΔPRP from S1 to S2 (*r* = −0.47, *p* = 0.035). MIIT induced a marked increase in PRP followed by a decrease during recovery. Changes in PPS and salivary orexin-A were not significantly correlated in the full MIIT sample. In the separate MIIT sub-analysis, a positive correlation was observed during the 30-min recovery period (*r* = 0.76, *p* = 0.029, and *n* = 8).

**Conclusion:**

MIIT produced a clear cardiovascular response, whereas MAST did not produce the expected immediate cardiovascular stress response. The MIIT sub-analysis supports a possible association between changes in salivary orexin-A and PPS during recovery but does not establish a causal or central orexin-mediated mechanism. Replication in a larger population using technically successful and standardized MIIT and MAST procedures is required.

## Introduction

Stress is a fundamental neurobiological mechanism enabling adaptive responses to environmental challenges (McEwen, 2007). Acute stress activates coordinated autonomic and neuroendocrine pathways, enhancing cardiovascular output, energy metabolism, and mental alertness (McEwen, 2007).

These responses involve rapid activation of the sympathetic autonomic nervous system together with neuroendocrine signaling through the hypothalamic-pituitary-adrenal (HPA) axis, enabling the organism to mobilize energy resources and behavioral readiness during demanding or threatening situations (Bankenahally and Krovvidi, 2016; Hellhammer et al., 2009). When followed by adequate recovery, such responses are adaptive as well as protective (McEwen, 2007; Ganzel et al., 2010). In a broader clinical perspective, stress-related autonomic dysregulation represents a major global health challenge, contributing substantially to the burden of cardiovascular disease, metabolic disorders and depression, and to reduced workability and quality of life worldwide (McEwen and Akil, 2020; Fink, 2016a).

However, prolonged activation of these stress pathways, accompanied by insufficient recovery may therefore impair physiological autonomic regulatory mechanisms and eventually lead to autonomic nervous system dysfunction (ANSD) and may contribute to the development of chronic stress-related conditions (Bankenahally and Krovvidi, 2016), as well as cardiovascular pathology, metabolic disturbances, and mental disorders (McEwen and Akil, 2020).

Despite the central role of stress in the pathophysiology of these and other contemporary diseases, the clinical assessment of stress still relies mostly on subjective self-report measures (Fink, 2016b). Physiological markers such as cortisol, blood pressure, and heart rate variability are influenced by multiple confounders, including medications such a beta-blockers (Lin et al., 1999) and do not consistently reflect the dynamics of central autonomic regulation, due to their peripheral location of recording ANS activity (McEwen and Akil, 2020). There is therefore a need for objective, accessible markers reflecting central autonomic stress integration (Selye, 1932). An ideal physiological marker should reflect rapid changes in autonomic regulation and provide insight into central autonomic mechanisms coordinating stress responses rather than only measuring downstream peripheral effects (Bankenahally and Krovvidi, 2016). The identification of such markers could potentially improve both experimental research on stress physiology and the clinical assessment of stress-related autonomic dysregulation in daily clinical practice.

The orexin (hypocretin) system, originating from the lateral hypothalamus and nearby perifornical and dorsomedial regions, plays a central role as a master regulator of autonomic activity, integrating arousal, stress responsiveness, metabolic state, cardiovascular control and homeostatic function (Chieffi et al., 2017). Orexin-A signaling projects to key brainstem nuclei including the locus coeruleus, dorsal raphe nucleus, and tuberomammillary nucleus, as well as to hypothalamic and limbic regions involved in cardiovascular regulation (Inutsuka and Yamanaka, 2013). Through these projections, orexin modulates sympathetic tone, cardiovascular activation, and behavioral arousal, and experimental studies have shown increased heart rate and blood pressure following orexin neuron activation (Chieffi et al., 2017; Tsujino and Sakurai, 2013; De Luca et al., 2022). In addition to its role in autonomic regulation, the orexin system responds to circadian rhythm, metabolic and emotional/mental stimuli, thereby enabling coordinated physiological and behavioral adaptive responses to environmental demands (Browning and Travagli, 2014).

Periosteal Pressure Sensitivity (PPS) at the sternum has been proposed as a simple non-invasive and objective physiological stress marker (Ballegaard et al., 2009). When professional opera singers were exposed to acute performance stress, it was shown that PPS correlated significantly with heart rate, blood pressure, and pressure-rate product, supporting its association with sympathetic activation (Ballegaard et al., 2009). PPS has also been found to reflect persistent stress load and autonomic imbalance (Ballegaard et al., 2014, 2015).

PPS has been associated with autonomic cardiovascular responses during tilt table testing in coronary artery disease. The baroreflex and HRV response to tilting appeared to modify the effect of beta-blocker treatment, while the PPS response remained unchanged. PPS has also been linked to disturbances in the autonomic homeostatic regulation of glucose metabolism and mood. This finding led to the hypothesis that PPS may reflect central autonomic function (Ballegaard et al., 2015).

The periosteum is densely innervated with nociceptive nerve endings and is highly responsive to mechanical stimulation (Ballegaard et al., 2009). Changes in the pressure sensitivity at the sternum may therefore reflect changes in nociceptive signaling associated with autonomic activation and deactivation (Ballegaard et al., 2009). The observation that beta-blockade did not affect PPS, combined with the involvement of the lateral hypothalamic orexin system in the homeostatic regulation of glucose metabolism, nociception and depression (Faber et al., 2021, 2023; Gjedde et al., 2023), led to the hypothesis of the present study: PPS and the orexin system may be associated through central autonomic homeostatic regulation. As such, the present study examined whether changes in salivary orexin-A were associated with changes in PPS and PRP during experimentally induced mental and physical stress and subsequent recovery in healthy females.

## Methods

### Study design

Baseline measurements (S1) included systolic blood pressure, heart rate, PRP, PPS, and salivary orexin-A, which were obtained prior to each experimental intervention to establish physiological starting conditions.

The study was conducted as a randomized cross-over trial. Each participant completed two experimental conditions separated by a washout period: the Maastricht Acute Stress Test (MAST) and a moderate-intensity interval training (MIIT) protocol. The order of the interventions was randomized, and each participant served as her own control.

A cross-over design was chosen to allow within-subject comparison of physiological responses to the two experimental tests, thereby reducing inter-individual variability in autonomic regulation and increasing sensitivity when examining short-term physiological responses to experimentally induced stressors.

The study was conducted at the Technical University of Denmark (DTU). Participants were allocated to one of two experimental sequences, MAST-first or MIIT-first, using coded randomization in Microsoft Excel. For each participant, the two experimental sessions were conducted approximately 1 week apart and at approximately the same time of day to reduce potential carryover effects and variations related to the time of day, respectively. Participant data and biological samples were pseudonymized using randomly generated identification codes.

The study was approved by the Regional Scientific Ethics Committee (VEK) and conducted in accordance with the Declaration of Helsinki. Written informed consent was obtained from all participants.

Sample size was estimated a priori using correlations reported by Ballegaard et al. (2009). Using Fisher's *z*-transformation, a two-sided significance level of 0.05, and 80% statistical power, approximately 25 participants were required to detect a correlation of *r* = 0.54, whereas approximately 14 participants were required to detect a correlation of *r* = 0.70. Thirty-six participants were recruited, of whom 20 fulfilled the eligibility criteria and completed both procedures.

The 30-min recovery measurement was selected based on Moscatelli et al. (2024), in which salivary orexin-A reached its highest measured concentration 30 mins after repetitive transcranial magnetic stimulation in female volleyball players. A measurement after 15 mins was included to assess an intermediate stage of recovery. As described in the protocol, the primary measurement intervals were the immediate response from S1 to S2 and the complete 30-min recovery response from S2 to S4. The 15-min recovery response from S2 to S3 was a secondary outcome.

### Participants

Twenty healthy female students aged 20–28 years were included. Restricting the cohort to young healthy females minimized biological variability related to sex differences and age-related changes in autonomic regulation.

A homogeneous study population was chosen to reduce potential confounding effects from age-related cardiovascular variability, chronic disease, or medication use that could influence the autonomic response.

Participants were instructed to avoid food, caffeine, alcohol, tobacco, and intense physical or mental exertion for at least 1 h prior to testing to minimize physiological fluctuations that could influence cardiovascular or hormonal measurements.

Participants were excluded at screening if their PPS value at the sternum was >60. The PPS threshold was based on Ballegaard et al. (2012), as values >60 were considered indicative of reduced resilience and a reduced capacity for appropriate ANS adaptation. Additional exclusion criteria included resting systolic blood pressure ≥140 mmHg, resting heart rate ≥100 beats per minute, Clinical Stress Score >8, Major Depression Inventory score >15, and WHO-5 score < 50.

Of the 36 potential participants recruited, 16 were excluded. The exclusions comprised medication use (*n* = 5), an acute medical condition (*n* = 1), withdrawal (*n* = 3), questionnaire scores outside the predefined screening limits (*n* = 5), and a screening PPS value above 60 (*n* = 2).

### Experimental procedures

All experimental sessions were conducted in a quiet environment under controlled ambient conditions to minimize external stressors. Participants rested for approximately 10 mins prior to baseline measurements to allow stabilization of cardiovascular parameters before the start of data collection.

Measurements were obtained at baseline after the initial resting period (S1), immediately after the experimental procedure (S2), after 15 mins of recovery (S3), and after 30 mins of recovery (S4).

#### Maastricht acute stress test (MAST)

The MAST consisted of cognitive stress combined with physical stress (cold water exposure), designed to increase sympathetic autonomic activity (Shilton et al., 2017).

The test combines elements of a cold-water immersion test with a mental arithmetic task performed under time pressure and evaluative conditions. Participants shifted between immersing their hand in 2°C cold water and completing challenging mental arithmetic calculations by serial subtraction (counting backwards from 2,043 in steps of 17) while a metronome provided time pressure, thereby inducing both physiological and psychological stress responses.

Previous studies have demonstrated that this protocol reliably activates sympathetic autonomic responses and increases subjective stress perception (Shilton et al., 2017).

#### Moderate-intensity interval training (MIIT)

The MIIT protocol consisted of short bursts of moderate-intensity bicycle exercise, designed to induce autonomic modulation and increased parasympathetic activity during recovery.

The exercise was performed on a stationary bicycle ergometer (TP200C Masterfit, Master Fitness/Abilica AS, Oslo, Norway). Participants cycled at a cadence of 70–100 revolutions per minute with the resistance initially set at 2.0 kilograms. The procedure comprised three 2-min cycling intervals separated by 2-min rest periods, giving a total duration of approximately 10 mins. The workload was adjusted to increase heart rate by approximately 30%−40%, to a maximum of approximately 150 beats per minute.

The protocol predefined that the MIIT should produce an increase in PRP greater than 10,000 mmHg × bpm from S1 to S2 to ensure the intended degree of ANS activation. The calculation underlying this threshold was based on Ballegaard et al. (2009). Eight participants did not achieve this predefined increase, leaving a technically correct MIIT subgroup of 12 participants. For transparency, results are presented for both the full sample (*n* = 20) and this subgroup (*n* = 12).

### Outcome measures

#### Periosteal Pressure Sensitivity (PPS)

PPS was measured using a digital algometric device (StressMeter, Nordic Heart Center Ltd., Denmark). The device applies gradually increasing pressure on the sternum until the participant reports the *first* sensation of pain or discomfort.

Measurements were obtained at the midline of the sternum where the periosteum can be stimulated with minimal interference from surrounding muscle tissue. The periosteum is richly innervated with nociceptive nerve endings, making it highly sensitive to mechanical stimulation.

Measurements were performed twice at the same location with a short interval between measurements. If the two readings differed by more than 10 units, a third measurement was performed. The final PPS value was calculated as the average of the two closest measurements.

The PPS measurement procedure followed previously described standardized protocols developed for assessing sternal PPS (Ballegaard et al., 2009). The algometric device allows controlled and reproducible pressure application, thereby reducing variability associated with manual stimulation.

Previous studies have reported acceptable intra-observer reproducibility of PPS measurements when performed using standardized procedures and repeated measurements. The use of duplicate measurements with predefined acceptance criteria was therefore applied to improve reliability of the recorded PPS values (Ballegaard et al., 2009).

#### Pressure Rate Product (PRP)

Was calculated as systolic blood pressure multiplied by heart rate and used as an indirect measure of cardiac workload. PRP reflects the amount of work performed by the heart and the myocardial oxygen demand, making it a useful indicator of cardiovascular strain during physiological stress or exercise (Wilkinson et al., 1979). In the present study, it was included to ensure an appropriate increase in workload during exercise (the MIIT test) as well as during the mental test (the MAST test) as found in the previous study by Ballegaard et al. (2009). Blood pressure was measured using an automated upper-arm monitor (Omron Healthcare Co., Ltd., 53 Kunotsubo, Terado-cho, Muko, Kyoto 617-0002, Japan).

#### Salivary orexin-A

Salivary orexin-A concentrations were quantified using a competitive enzyme-linked immunosorbent assay (Human OXA/Orexin-A ELISA Kit, Cat. No. E-EL-H1015, Elabscience, Wuhan, China). The assay had a manufacturer-reported analytical sensitivity of 37.5 pg/mL and a detection range of 62.5–4,000 pg/mL. The manufacturer-reported intra-assay coefficients of variation ranged from 5.04% to 6.63%, and the inter-assay coefficients of variation ranged from 6.02% to 8.30%. The reported recovery range was 80%−120%. Saliva samples were collected using sterile cotton swabs at each predefined time point and stored at −20 °C until analysis. Before analysis, samples were thawed and centrifuged at 1,000 × g for 20 mins at 2–8 °C. Orexin-A concentrations were determined according to the manufacturer's protocol.

Samples were intended to be analyzed in triplicate. However, because of limited saliva volume and the loss of assay wells following a technical laboratory error, some samples were analyzed in triplicate and others in duplicate. Results from assay wells affected by the technical error were discarded. Study-specific intra- and inter-assay coefficients of variation were not calculated, which limits the assessment of analytical precision in the present study.

Orexin-A measurements were not available for all participants at all time points. During MIIT, orexin-A data were available for 17 participants at S1, 15 at S2, 14 at S3, and 14 at S4. During MAST, orexin-A data were available for 15 participants at each time point. Analyses involving orexin-A were based on participants with complete observations for the specific interval analyzed. Samples with insufficient saliva volume were treated as missing and were not substituted.

### Statistical analysis

All statistical analyses were performed using standard statistical software [IBM SPSS Statistics (IBM Corp., Armonk, NY, USA) and Microsoft Excel (Microsoft Corporation, Redmond, WA, USA)]. Prior to correlation analysis, variables were inspected for distributional characteristics and potential outliers to ensure appropriate interpretation of parametric correlation coefficients.

Changes between the predefined time points were analyzed using paired-samples *t*-tests. Pearson correlation coefficients were used to examine associations between changes in PPS, PRP, and salivary orexin-A. Changes were calculated separately for the exercise or stress phase (ΔS2–S1), early recovery (ΔS3–S2), and the complete 30-min recovery period (ΔS4–S2). According to the protocol, ΔS2–S1 and ΔS4–S2 were the primary intervals, whereas ΔS3–S2 was a secondary interval. MIIT analyses were performed separately for the full sample (*n* = 20) and the subgroup fulfilling the protocol-defined PRP response criterion (*n* = 12).

Missing observations were not substituted or imputed. All analyses were based on observed data only. Analyses involving orexin-A included only participants with complete paired observations for the relevant variables and interval. The exact number of participants included is reported in each table.

No formal correction for multiple comparisons was applied because the comparisons represented separately defined intervention and recovery periods and were used to examine the predefined physiological response patterns. However, several paired comparisons and correlations were performed. Exact *p*-values are therefore reported, and secondary findings and findings from the separate MIIT sub-analysis are interpreted cautiously. A two-sided *p*-value < 0.05 was considered statistically significant.

## Results

### Participant flow and baseline characteristics

Of the 36 recruited potential participants, 16 were excluded based on the exclusion criteria. The remaining 20 participants were enrolled in the study and completed both experimental procedures (MAST and MIIT) in a randomized crossover design. Participant flow throughout the study is illustrated in Figure 1.

**Figure 1:**
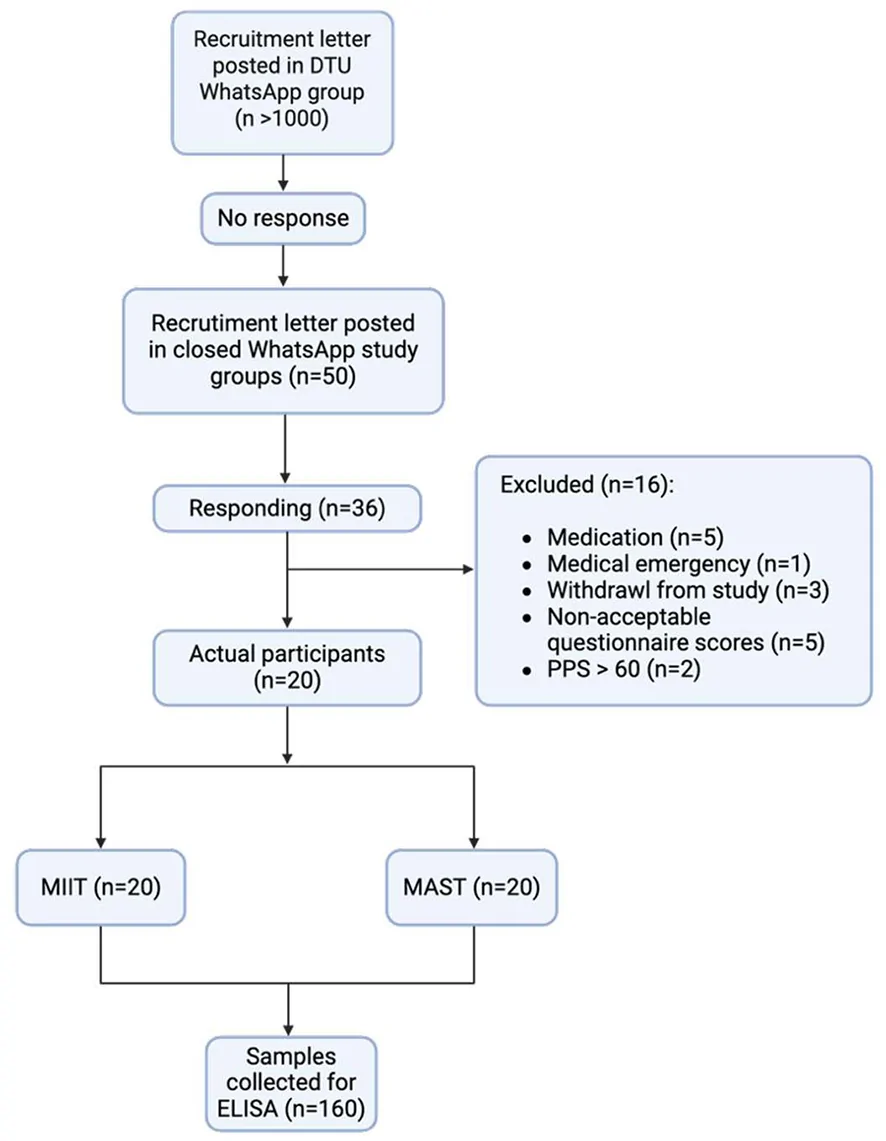
Consort diagram illustrating participant flow through the study.

The 16 exclusions comprised medication use (*n* = 5), an acute medical condition (*n* = 1), withdrawal (*n* = 3), questionnaire scores outside the predefined screening limits (*n* = 5), and a screening PPS value above 60 (*n* = 2).

PPS and PRP data were available for all 20 participants at all four time points. Some orexin-A measurements were missing because of insufficient saliva volume or technical limitations. Missing observations were not substituted.

### MAST

In the MAST condition (*n* = 20), PPS showed no significant changes from baseline to post-stress and recovery (all *p* > 0.05). PRP decreased significantly during early recovery from S2 to S3 (ΔPRP S3–S2: −879 ± 1,833 mmHg × bpm, *p* = 0.045), while the remaining PRP changes were non-significant. Salivary orexin-A showed no significant changes during any interval.

A significant negative correlation was observed between ΔPPS and ΔPRP from S1 to S2 (*r* = −0.47, *p* = 0.035, and *n* = 20). Results across all time points are presented in Table 1.

**Table 1:** PPS, PRP, and salivary orexin-A in the MAST condition (*n*= 20).

Outcome	S1	S2	S3	S4	ΔS2–S1; *p*	ΔS3–S2; *p*	ΔS4–S2; *p*
	Baseline	Post-stress	Recovery (15 min)	Recovery (30 min)			
PPS	53.48 ± 12.02 (20)	55.92 ± 15.2 (20)	52.40 ± 14.82 (20)	51.88 ± 14.34 (20)	2.45 ± 7.81 (20); 0.177	−3.53 ± 8.07 (20); 0.066	−4.05 ± 9.62 (20); 0.075
PRP (mmHg × bpm)	6,823 ± 1,321(20)	7295 ± 2,963 (20)	6,416 ± 1,929 (20)	6,804 ± 1,690 (20)	473 ± 2,818 (20); 0.462	**−879** **±1,833 (20); 0.045**	−492 ± 2,197 (20); 0.329
Orexin-A (pg/mL)	48.66 ± 13.80 (15)	54.41 ± 23.77 (15)	55.03 ± 19.47 (15)	53.87 ± 18.41 (15)	5.75 ± 20.66 (15); 0.299	0.62 ± 19.28 (15); 0.903	−0.54 ± 12.88 (15); 0.873

Values at S1–S4 and changes are presented as mean ± SD (*n*). *p*-values for changes between time points were calculated using paired-samples t-tests. The significant results are shown in bold.

### MIIT

In the full MIIT sample, PRP increased significantly from S1 to S2 and decreased significantly from S2 to S3 and from S2 to S4 (all *p* < 0.001). PPS and salivary orexin-A did not change significantly during any of the predefined intervals.

### Separate MIIT sub-analysis

In the separate MIIT sub-analysis (*n* = 12), PPS decreased significantly during exercise from S1 to S2 and increased significantly during the complete 30-min recovery period from S2 to S4. PRP increased significantly during exercise and decreased significantly during recovery, while salivary orexin-A did not change significantly.

### Correlations

In the full MIIT sample, no significant correlations were observed between changes in PPS and changes in PRP or salivary orexin-A. A significant positive correlation was observed between ΔPRP and Δorexin-A during the first 15 mins of recovery (S3–S2: *r* = 0.61, *p* = 0.026, and *n* = 13).

In the separate MIIT sub-analysis, a strong positive correlation was observed between ΔPPS and Δorexin-A during the complete 30-min recovery period (S4–S2: *r* = 0.76, *p* = 0.029, and *n* = 8; Figure 2). The corresponding correlation during exercise was moderate and positive but non-significant (S2–S1: *r* = 0.47, *p* = 0.171, and *n* = 10). The correlation between ΔPRP and Δorexin-A during early recovery was negative and close to, but did not reach, statistical significance (S3–S2: *r* = −0.63, *p* = 0.051, and *n* = 10). Results for the full MIIT sample, the protocol-defined subgroup, and the correlation analyses are presented in Tables 2–4.

**Figure 2:**
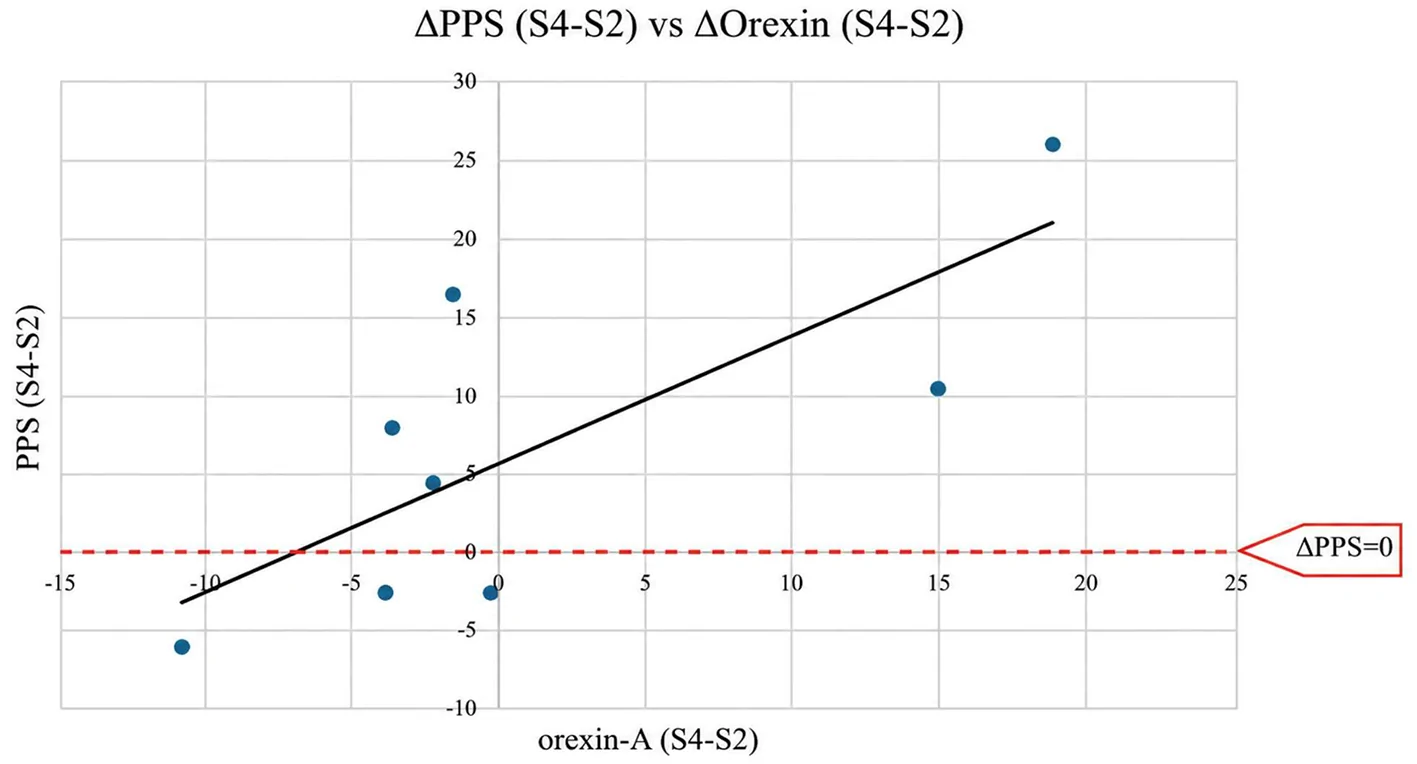
Association between changes in PPS and salivary orexin-A during MIIT in the subgroup during the 30-min recovery period (S4–S2; *n*= 8; *r*= 0.76; *p*= 0.029). The red dashed horizontal line indicates ΔPPS = 0. Pearson correlations were based on participants with complete paired data. The sub-analysis included participants who achieved the predefined ΔPRP > 10,000 mmHg × beats/min (*n*= 12).

**Table 2:** PPS, PRP, and salivary orexin-A during MIIT in the full sample.

Outcome	S1	S2	S3	S4	ΔS2–S1; *p*	ΔS3–S2; *p*	ΔS4–S2; *p*
	Baseline	Post-stress	Recovery (15 min)	Recovery (30 min)			
PPS	53.35 ± 12.77 (20)	50.48 ± 10.25 (20)	51.67 ± 9.91 (20)	53.30 ± 12.92 (20)	−2.88 ± 10.05 (20); 0.216	1.20 ± 5.41 (20); 0.334	2.83 ± 10.67 (20); 0.251
PRP (mmHg × bpm)	6,534 ± 1,253 (20)	16,730 ± 5,275 (20)	8,220 ± 1,725 (20)	7,190 ± 1,758 (20)	**10,197** **±5,152 (20);** ** < 0.001**	**−8,510** **±4,445 (20);** ** < 0.001**	**−9,540** **±4,436 (20);** ** < 0.001**
Orexin-A (pg/mL)	50.39 ± 21.84 (17)	50.38 ± 12.26 (15)	56.77 ± 29.49 (14)	49.97 ± 16.39 (14)	0.07 ± 22.33 (15); 0.991	8.23 ± 30.13 (13); 0.344	1.72 ± 11.36 (12); 0.611

Values at S1–S4 and changes are presented as mean ± SD (*n*). *p*-values for changes between time points were calculated using paired-samples t-tests. The significant results are shown in bold.

**Table 3:** PPS, PRP, and salivary orexin-A during MIIT in participants fulfilling the protocol-defined response criterion.

Outcome	S1	S2	S3	S4	ΔS2–S1; *p*	ΔS3–S2; *p*	ΔS4–S2; *p*
	Baseline	Post-stress	Recovery (15 min)	Recovery (30 min)			
PPS	56.58 ± 11.69 (12)	51.58 ± 10.15 (12)	53.46 ± 9.84 (12)	57.42 ± 11.22 (12)	**−5.00** **±7.18 (12); 0.034**	1.88 ± 5.09 (12); 0.228	5.83 ± 8.82 (12); 0.043
PRP (mmHg × bpm)	6,598 ± 1,488 (12)	20,184 ± 1,393 (12)	8,788 ± 1,528 (12)	7,909 ± 1,648 (12)	**13,586** **±2.19 (12);** ** < 0.001**	**−11,396** **±2.7 (12);** ** < 0.001**	−12,275 ± 2.21 (12); < 0.001
Orexin-A (pg/mL)	48.17 ± 13.73 (12)	51.52 ± 11.06 (10)	47.94 ± 13.73 (11)	50.98 ± 17.96 (10)	3.91 ± 15.84 (10); 0.455	−2.23 ± 8.99 (10); 0.453	1.45 ± 10.10 (8); 0.697

Values at S1–S4 and changes are presented as mean ± SD (*n*). *p*-values for changes between time points were calculated using paired-samples t-tests. The significant results are shown in bold.

**Table 4:** Pearson correlations between changes in PPS, PRP, and salivary orexin-A during MAST and MIIT.

Type of test	Association	Interval	*n*	Pearson's, *r*	*p*-value
MAST, full sample (*n* = 20)	ΔPPS vs. ΔPRP	S2–S1	20	**−0.47**	**0.035**
		S3–S2	20	−0.33	0.151
		S4–S2	20	−0.24	0.304
	ΔPPS vs. Δorexin-A	S2–S1	15	0.17	0.539
		S3–S2	15	0.14	0.621
		S4–S2	15	−0.07	0.761
	ΔPRP vs. Δorexin-A	S2–S1	15	−0.24	0.383
		S3–S2	15	−0.20	0.464
		S4–S2	15	−0.08	0.735
MIIT, full sample (*n* = 20)	ΔPPS vs. ΔPRP	S2–S1	20	0.05	0.842
		S3–S2	20	0.09	0.705
		S4–S2	20	−0.29	0.217
	ΔPPS vs. Δorexin-A	S2–S1	15	0.14	0.619
		S3–S2	13	−0.17	0.575
		S4–S2	12	0.33	0.288
	ΔPRP vs. Δorexin-A	S2–S1	15	0.24	0.393
		S3–S2	13	**0.61**	**0.026**
		S4–S2	12	0.32	0.305
MIIT, sub-group (*n* = 12)	ΔPPS vs. ΔPRP	S2–S1	12	0.37	0.242
		S3–S2	12	0.49	0.109
		S4–S2	12	0.19	0.549
	ΔPPS vs. Δorexin-A	S2–S1	10	0.47	0.171
		S3–S2	10	−0.25	0.493
		S4–S2	8	**0.76**	**0.029**
	ΔPRP vs. Δorexin-A	S2–S1	10	0.52	0.127
		S3–S2	10	−0.63	0.051
		S4–S2	8	0.23	0.576

The significant results are shown in bold.

## Discussion

During the MIIT-test, orexin-A levels did not change significantly across time points. However, in the protocol-defined MIIT subgroup, changes in PPS and salivary orexin-A were positively and significantly correlated during the 30-min recovery period. This finding supports the original hypothesis that the orexin receptor system and PPS dynamics may be associated with physiological autonomic adaptation. No corresponding significant association was observed in the full MIIT sample.

Orexin neurons in the lateral hypothalamus project to several brain regions involved in autonomic regulation, including the locus coeruleus and other brainstem nuclei associated with cardiovascular control. Activation of the orexin system has been shown to increase sympathetic activity, heart rate, and blood pressure.

The significant correlation between Δorexin-A and ΔPPS observed in the protocol-defined subgroup, during the recovery phase, may indicate that orexin-PPS related mechanisms are involved in the physiological adaptive processes that occur during and following acute moderate physical stress. However, this interpretation remains exploratory. The adaptive autonomic response during moderate physical exercise involves acute stress-induced coordinated and reciprocal changes in autonomic homeostatic regulation, including regulation of cardiovascular workload and the physiological balance between the sympathetic and parasympathetic branches of the autonomic nervous system (Goldstein, 2021). The association observed in the present study suggests that orexin-A and PPS dynamics may be associated with these regulatory processes.

### PPS and PRP in previous studies

Previous cross-over studies have demonstrated a link between PPS and cardiovascular activation, measured as PRP in during both mental stress and in moderate physical exercise testing (Ballegaard et al., 2009), suggesting that PPS may serve as a peripheral marker of stress-related autonomic activation. In a randomized cross-over study of professional opera singers exposed to performance stress, PPS increased in parallel with cardiovascular activation and correlated positively with PRP (Ballegaard et al., 2009). Similar findings were later observed in opera students during audition settings, in which mental performance stress evoked coordinated increases in PPS and cardiovascular parameters.

### Orexin-A and PPS

The positive and significant correlation between changes in PPS and salivary orexin-A during the 30-min recovery period supports the hypothesis of an association between PPS and orexin. The correlation during exercise was positive but non-significant. The length of the recovery period was selected based on Moscatelli et al. (2024), in which salivary orexin-A reached its highest measured concentration 30 mins after repetitive transcranial magnetic stimulation in female volleyball players.

The absence of a significant mean change in salivary orexin-A does not contradict the observed correlation. The paired analysis determines whether the mean change in the group differs from zero, whereas the correlation analysis determines whether participants with larger changes in one variable also demonstrate larger changes in the other variable. Due to the small sample size, the results should be repeated independently in a larger sample size study.

Although a correlation coefficient of 0.76 represents a strong association (Mukaka, 2012), its clinical significance cannot be determined by a single study and needs repetition, preferably in a larger study.

While saliva sampling provides a practical and non-invasive approach for human studies, the relationship between peripheral orexin levels and central neuronal activity remains incompletely understood. Salivary orexin-A cannot be considered a direct measure of central orexin activity. The association may reflect central orexin signaling, peripheral regulation, or another physiological mechanism affecting both measures. To date, studies directly comparing salivary orexin-A with matched blood or cerebrospinal fluid orexin-A concentrations appear to be lacking. One way to get closer to the answer to this question is to compare blood and salivary orexin, a potential task for a future study.

### Why did MAST not reproduce performance stress findings?

The lack of significant effects following the MAST differs from earlier performance-based stress studies (Shilton et al., 2017). One possible explanation is that the laboratory stress tasks used in the MAST may not evoke the same emotional intensity, social evaluative threat, or personal relevance in engineering students at the Technical University of Denmark as in the population groups previously exposed to the MAST test.

Furthermore, the previous PPS-related studies used different forms of mental stress induction, such as public performance and auditions (Ballegaard et al., 2009). Solo opera performances and auditions likely trigger stronger limbic–hypothalamic activation than the standardized MAST procedure used in this study (Ballegaard et al., 2009). Consequently, different stress protocols may produce varying degrees of autonomic activation. The non-significant and small physiological changes observed in this study may therefore indicate that the stress response induced in this controlled laboratory setting was limited. In addition, the relatively small sample size used in this study may reduce the statistical power to detect small physiological changes.

It should also be taken into consideration that the participants in the present study were healthy young females who may not have experienced strong emotional engagement but rather liked the challenge associated with the experimental task. Psychological stress responses are strongly influenced by perceived relevance and emotional involvement. Laboratory protocols that lack personal significance may therefore evoke weaker physiological responses compared with real-life performance situations.

Furthermore, the timing of neuroendocrine responses may differ from the more immediate cardiovascular responses observed during acute stress. While cardiovascular variables such as heart rate and blood pressure can change rapidly, hormonal responses may occur over longer time scales (Hellhammer et al., 2009) or require stronger emotional activation to produce measurable changes. It is therefore possible that the MAST protocol used in this study did not generate sufficient physiological activation to induce detectable changes in salivary orexin-A concentrations, PRP or PPS.

### Strengths and limitations

A major strength in this study is the randomized cross-over design, minimizing inter-individual variability by having each participant serve as her own control. Measuring PPS, PRP, and orexin-A at the same time made it possible to examine how cardiovascular and neuroregulatory responses co-vary. The use of a homogeneous group of healthy young women reduced biological variation and improved internal consistency.

However, several limitations should be noted. The sample size was small, and the inclusion of orexin-A measurements was exploratory. Salivary orexin-A is an indirect measure and does not allow direct conclusions about central orexin activity.

An additional limitation concerns the technical aspects of saliva collection. Some participants produced insufficient saliva volumes for complete orexin-A analysis, resulting in missing data and reduced sample sizes in certain analyses.

Missing observations were not substituted or imputed. Only observed data were included, and the exact number of participants contributing to each analysis is reported in the tables. This approach increases transparency but reduced the statistical power of analyses involving salivary orexin-A.

In some cases, the limited sample volume precluded duplicates or triplicate ELISA measurements, potentially reducing analytical precision. Given that reduced salivary secretion can accompany sympathetic activation (Frederiksen et al., 2024), this observation may suggest the presence of an autonomic stress response despite the absence of significant changes in the measured physiological variables. However, as salivary flow and subjective dryness were not systematically assessed, this interpretation remains speculative.

A key limitation is that the MAST protocol did not produce the expected sufficient sympathetic activation, as PRP did not increase significantly from baseline to post-stress. This indicates that the MAST test was insufficient as a sympathetic stressor in this sample, which may reflect individual variation in stress responsiveness, methodological factors, or characteristics specific to the participant group. Consequently, hypothesis 1 remains untested rather than rejected, as the absence of a valid stress response means that the role of orexin-A in acute mental stress could not be evaluated from the present data. Future studies should consider optimizing the implementation of the MAST protocol and/or increasing the sample size to ensure adequate sympathetic activation.

The technical execution of MIIT also varied between participants, as eight of the 20 participants did not achieve the response required by the protocol. Although the response criterion was predefined, the decision to report the participants who fulfilled it as a separate subgroup was made during the subsequent analysis phase. Presenting both the full sample and the subgroup increases transparency, but the subgroup analysis included only eight complete paired observations for the significant correlation and may therefore be sensitive to individual observations.

Several paired comparisons and correlations were also examined without formal adjustment for multiple comparisons. The subgroup finding should consequently be interpreted with caution until it has been independently replicated.

Additionally, the study population consisted exclusively of young healthy females, which improves internal validity but may limit generalizability of the findings and therefore, similar studies should be done on other healthy populations, including males and older individuals.

### Future studies

Previous studies have demonstrated that PPS is sensitive to both acute and persistent stress and correlates with cardiovascular and autonomic parameters across a broad range of populations and stress contexts, including mental performance stress, physical exercise, cardiovascular disease, diabetes, and depression (Ballegaard et al., 2009, 2014, 2015, 2023; Ørsted et al., 2025; Hecquet et al., 2024). Future studies should therefore build on this evidence by examining whether PPS, in combination with orexin-A, can provide additional insight into the mechanisms underlying stress-related autonomic regulation, and thus potentially elucidate possible physiological and biochemical discrimination points when comparing and distinguishing between transient and persistent stress.

Additionally, replication of these findings in a larger population would help determine whether the relationship between PPS and orexin remains consistent across different types and intensities of stress. Measuring orexin-A in plasma may help clarify whether the observed effects reflect systemic or peripheral processes and may help evaluate the clinical usability of salivary orexin-A assessments. Including additional autonomic measures, such as heart rate variability, could further improve understanding of stress regulation.

The study should be repeated with a larger number of participants and technically standardized MIIT and MAST procedures. MIIT should reliably produce the predefined cardiovascular response, while MAST should be optimized to ensure sufficient sympathetic activation.

Future research should also explore the timing of orexin signaling during stress exposure and recovery, as well as its interaction with other neuroendocrine systems involved in stress regulation. Investigating these relationships may help clarify the mechanisms linking central stress signaling with peripheral physiological markers such as PPS.

### Clinical perspectives

An important observation of the present study is that moderate physical stress was associated with a decrease in PPS during exercise followed by coordinated recovery dynamics. In contrast to mental stress tasks, the short cycling intervention appeared to induce autonomic changes that returned to baseline rather than prolonged sympathetic activation.

PPS has been proposed as a clinically relevant indicator of autonomic nervous system activity and dysfunction, with potential applications as both a diagnostic tool and a therapeutic target (Ørsted et al., 2025; Salvini et al., 2023). If future studies confirm the present findings that PPS reflects aspects of autonomic homeostatic regulation, this measurement may provide a simple method for assessing physiological responses to stress and recovery. Further research is needed to determine whether changes in PPS can serve as a useful marker of autonomic stress responses in different physiological and clinical conditions.

## Conclusion

In this randomized cross-over study, conducted in healthy female students, moderate-intensity interval physical exercise elicited robust cardiovascular activation. In the separate MIIT sub-analysis, changes in salivary orexin-A were significantly correlated with changes in PPS during the 30-min recovery period.

Although the study was limited by the small sample size and the reduced number of complete salivary orexin-A observations, the findings support the original study hypothesis and thus support further investigation of the relationship between salivary orexin-A, PPS, and autonomic responses during physical exercise and recovery. Replication in a larger population is required before the physiological or clinical significance of the observed association can be determined.

## Data Availability

The datasets generated and analysed during the current study are available from the corresponding author upon reasonable request. Data sharing may be subject to institutional guidelines and ethical considerations related to participant confidentiality.

## References

[B1] Ballegaard S. Bergmann N. Karpatschof B. Kristiansen J. Gyntelberg F. Arendt-Nielsen L. . (2015). Association between pressure pain sensitivity and autonomic function as assessed by a tilt table test. Scand. J. Clin. Lab. Invest. 75, 345–354. doi: 10.3109/00365513.2015.1028095 25833816

[B2] Ballegaard S. Faber J. Selmer C. Gyntelberg F. Kreiner S. Karpatschof B. . (2023). In ischemic heart disease, reduced sensitivity to pressure at the sternum accompanies lower mortality after five years: evidence from a randomized controlled trial. J. Clin. Med. 12:7585. doi: 10.3390/jcm12247585 38137654 PMC10744062

[B3] Ballegaard S. Karpatschof B. Trojaborg W. Hansen A. M. Magnusson G. Petersen P. B. . (2009). A simple and objective marker for stress. Scand. J. Clin. Lab. Invest. 69, 713–721. doi: 10.3109/00365510903042734 19544223

[B4] Ballegaard S. Petersen P. B. Gyntelberg F. Faber J. (2012). The association between pressure pain sensitivity, and answers to questionnaires estimating psychological stress level in the workplace: a feasibility study. Scand. J. Clin. Lab. Invest. 72, 459–466. doi: 10.3109/00365513.2012.695023 22974296

[B5] Ballegaard S. Petersen P. B. Harboe G. S. Karpatschof B. Gyntelberg F. Faber J. . (2014). The association between changes in pressure pain sensitivity and changes in cardiovascular physiological factors associated with persistent stress. Scand. J. Clin. Lab. Invest. 74, 116–125. doi: 10.3109/00365513.2013.862847 24313546

[B6] Bankenahally R. Krovvidi H. (2016). Autonomic nervous system: anatomy, physiology, and relevance in anaesthesia and critical care medicine. BJA Educ. 16, 381–387. doi: 10.1093/bjaed/mkw011

[B7] Browning K. N. Travagli R. A. (2014). Central nervous system control of gastrointestinal motility and secretion and modulation of gastrointestinal functions. Compr. Physiol. 4, 1339–1368. doi: 10.1002/j.2040-4603.2014.tb00587.x PMC4858318 25428846

[B8] Chieffi S. Carotenuto M. Monda V. Valenzano A. Villano I. Precenzano F. . (2017). Orexin system: the key for a healthy life. Front. Physiol. 8:357. doi: 10.3389/fphys.2017.00357 PMC5450021 28620314

[B9] De Luca R. Nardone S. Grace K. P. Venner A. Cristofolini M. Bandaru S. S. . (2022). Orexin neurons inhibit sleep to promote arousal. Nat. Commun. 13:4163. doi: 10.1038/s41467-022-31591-y 35851580 PMC9293990

[B10] Faber J. Ballegaard S. Ørsted N. Eldrup E. Karpatschof B. Gyntelberg F. . (2023). In Type 2 Diabetes Mellitus, normalization of hemoglobin A1c accompanies reduced sensitivity to pressure at the sternum. Front. Neurosci. (2023) 17:1067098. doi: 10.3389/fnins.2023.1067098 37389368 PMC10303981

[B11] Faber J. Eldrup E. Selmer C. Pichat C. Hecquet S. K. Watt T. . (2021). Reduction of pressure pain sensitivity as novel non-pharmacological therapeutic approach to type 2 diabetes: a randomized trial. Front. Neurosci. (2021) 15:613858. doi: 10.3389/fnins.2021.613858 33776633 PMC7991917

[B12] Fink G. (2016a). Eighty years of stress. Nature 539, 175–176. doi: 10.1038/nature20473 27783596

[B13] Fink G. (2016b). Stress: Concepts, Cognition, Emotion, and Behavior: Handbook of Stress Series, Vol. 1. London: Academic Press.

[B14] Frederiksen E. H. Ramirez M. V. R. Moreno-Hay I. Miller C. S. Carlson C. R. (2024). Whole unstimulated salivary flow rate decreases during acute stressful condition. Oral Surg. Oral Med. Oral Pathol. Oral Radiol. 137, 594–603. doi: 10.1016/j.oooo.2024.02.028 38521649

[B15] Ganzel B. L. Morris P. A. Wethington E. (2010). Allostasis and the human brain: integrating models of stress from the social and life sciences. Psychol. Rev. 117:134. doi: 10.1037/a0017773 20063966 PMC2808193

[B16] Gjedde A. Bibby B. Gyntelberg F. Karpatschof B. Ballegaard S. Hjalmarson A. . (2023). Novel approach to depression in ischemic heart disease: attenuation of autonomic dysfunction lowers depression scores.

[B17] Goldstein D. S. (2021). Stress and the “extended” autonomic system. Auton. Neurosci. 236:102889. doi: 10.1016/j.autneu.2021.102889 34656967 PMC10699409

[B18] Hecquet S. K. Ballegaard S. Eldrup E. Hansen C. S. Hansen T. W. Harboe G. S. . (2024). New diabetic treatment by alleviation of autonomic nervous system dysfunction measured as periosteal pressure sensitivity at sternum improves empowerment, treatment satisfaction, and self-reported health of people with type 2 diabetes: a randomized trial. Diabetes Metab. Syndrome Obes. 17, 2519–2531. doi: 10.2147/DMSO.S455216 PMC11193981 38910915

[B19] Hellhammer D. H. Wüst S. Kudielka B. M. (2009). Salivary cortisol as a biomarker in stress research. Psychoneuroendocrinology 34, 163–171. doi: 10.1016/j.psyneuen.2008.10.026 19095358

[B20] Inutsuka A. Yamanaka A. (2013). The physiological role of orexin/hypocretin neurons in the regulation of sleep/wakefulness and neuroendocrine functions. Front. Endocrinol. 4:18. doi: 10.3389/fendo.2013.00018 PMC3589707 23508038

[B21] Lin J. L. Chan H. L. Du C. C. Lin I. N. Lai C. W. Lin K. T. . (1999). Long-term beta-blocker therapy improves autonomic nervous regulation in advanced congestive heart failure: a longitudinal heart rate variability study. Am. Heart J. 137, 658–665. doi: 10.1016/S0002-8703(99)70219-X 10097226

[B22] McEwen B. S. (2007). Physiology and neurobiology of stress and adaptation: central role of the brain. Physiol. Rev. 87, 873–904. doi: 10.1152/physrev.00041.2006 17615391

[B23] McEwen B. S. Akil H. (2020). Revisiting the stress concept: implications for affective disorders. J. Neurosci. 40, 12–21. doi: 10.1523/JNEUROSCI.0733-19.2019 PMC6939488 31896560

[B24] Moscatelli F. Monda A. Messina A. Monda M. Monda V. Villano I. . (2024). Evaluation of orexin-A salivary levels and its correlation with attention after non-invasive brain stimulation in female volleyball players. Sports Med. Open 10:32. doi: 10.1186/s40798-024-00698-5 PMC10994895 38573446

[B25] Mukaka M. M. (2012). A guide to appropriate use of correlation coefficient in medical research. Malawi Med. J. 24, 69–71. PMC3576830 23638278

[B26] Ørsted N. Ballegaard S. Kristiansen J. Gyntelberg F. Hjalmarson A. Faber J. (2025). Periosteal pressure sensitivity of the chest bone as a measure for autonomic function in ischemic heart disease. Front. Neurosci. 19:1574942. doi: 10.3389/fnins.2025.1574942 40470291 PMC12133862

[B27] Salvini V. Accioli R. Lazzerini P. E. Acampa M. (2023). Editorial: New challenges and future perspectives in autonomic neuroscience. Front. Neurosci. 17:1271499. doi: 10.3389/fnins.2023.1271499 PMC10482394 37680971

[B28] Selye H. (1932). Syndrome produced by diverse nocuos agents. Nature 32:138. doi: 10.1038/138032a0 9722327

[B29] Shilton A. L. Laycock R. Crewther S. G. (2017). The Maastricht Acute Stress Test (MAST): physiological and subjective responses in anticipation, and post-stress. Front. Psychol. 8:567. doi: 10.3389/fpsyg.2017.00567 PMC5395611 28469586

[B30] Tsujino N. Sakurai T. (2013). Role of orexin in modulating arousal, feeding, and motivation. Front. Behav. Neurosci. 7:28. doi: 10.3389/fnbeh.2013.00028 23616752 PMC3629303

[B31] Wilkinson P. L. Moyers J. R. Ports T. Chatterjee K. Ullyott D. Hamilton W. K. . (1979). Rate-pressure product and myocardial oxygen consumption during surgery for coronary artery bypass. Circulation 60, 170–173. doi: 10.1161/01.CIR.60.2.170 312708

